# Response to: Comment on Rohrscheib *et al*. 2016 "Intensity of mutualism breakdown is determined by temperature not amplification of *Wolbachia* genes"

**DOI:** 10.1371/journal.ppat.1006521

**Published:** 2017-09-11

**Authors:** Chelsie E. Rohrscheib, Francesca D. Frentiu, Emilie Horn, Fiona K. Ritchie, Bruno van Swinderen, Michael W. Weible, Scott L. O’Neill, Jeremy C. Brownlie

**Affiliations:** 1 School of Natural Sciences, Griffith University, Nathan, Australia; 2 Griffith Research Institute for Drug Discovery, Griffith University, Nathan, Australia; 3 Institute of Health and Biomedical Innovation and School of Biomedical Sciences, Queensland University of Technology, Kelvin Grove, Australia; 4 Queensland Brain Institute, The University of Queensland, St. Lucia, Australia; 5 School of Biological Sciences, Monash University, Clayton, VIC, Australia; 6 Environmental Futures Research Institute, Griffith University, Nathan, Australia; Stanford University, UNITED STATES

Unlike other intracellular bacteria, *Wolbachia* genomes are highly labile largely due to the presence of repetitive sequences such as transposons, active phage and high rates of recombination [1–3]. Within the *w*MelPop genome a locus of considerable instability, referred to as the Octomom locus, has been described by several studies [4–8]. As this locus is one of the few variable loci between *w*Mel (single Octomom locus, low bacterial density and non-pathogenic) and *w*MelPop (3–12 Octomom loci, high bacterial density and pathogenic) genomes, and no genetic transformation tools are available, there is considerable interest in understanding the link between genotype and phenotype. Chrostek and Teixeira hypothesized “that Octomom region amplification underlies *w*MelPop virulence” and went on to correlate increased Octomom copy number to increased bacterial density within the host and strength of pathology [5]. Absent from this study was a systematic assessment of temperature, which previous studies had shown to affect *w*MelPop pathology, and its effect on *w*MelPop density and Octomom copy number. We hypothesized that temperature would affect Octomom copy number and/or gene expression, and that such changes would affect the strength of pathology [8]. Our study showed that while Octomom copy number did vary over developmental time, no consistent trend was observed among Octomom copy number, bacterial density nor pathology [8]. We determined that temperature, not Octomom copy number or bacterial density, had the greatest effect on *w*MelPop infected host lifespan. The link between Octomom copy number and pathology was further challenged by our discovery of a pathogenic *Wolbachia* strain (*w*Mel3562) that maintained a single copy of the Octomom locus, and supports an earlier study of a *w*MelPop variant that lacks the Octomom locus but which is still pathogenic [7–9]. These observations suggest that at the very least, genetic elements beyond the Octomom locus are responsible for pathology.

Chrostek and Teixeira’s comment does not critique a major finding of our studies: that *w*MelPop density and rate of growth within the host was biphasic. That is, above a threshold temperature *w*MelPop rapidly established a high density within the host, while below that threshold *w*MelPop established a moderate density within the host at a slower rate. Importantly we showed that neither the rate or density of *w*MelPop had an effect on pathogenicity. As Chrostek and Teixeria make no mention of these results in their commentary we assume that they agree with our conclusion that neither absolute density nor the rate of growth alone affects the strength of pathology.

A major criticism of our study was the design of the qPCR experiment used to estimate Octomom copy number, in particular the absence of any reference to a calibrator. In their commentary, Chrostek and Teixeira suggest that “a calibrator with known Octomom copy number is required”, one such calibrator would be *w*MelCS which contains a single copy of the Octomom locus. Re-applying our qPCR assay, and analysing the data using the Pfaffl method, we estimated a 1:1 ratio of Octomom to *w*MelCS genome (S1 Fig; S1 Data). Consequently, we conclude that our qPCR assay was valid and our previous estimates of Octomom copy number correct.

In the Chrostek and Teixeira 2015 paper a selection experiment was made on the basis of Octomom copy number and not pathology. We concede that our description of Chrostek and Teixeira’s selection experiment was incorrect, however our criticism of their selection experiment remains: the design of the experiment was fundamentally flawed as it does not correct for selection upon the host. In the absence of these controls, no conclusions regarding Octomom copy number and phenotype can be inferred. The need for such controls has been highlighted by a previous selection study which sought to manipulate the strength of *w*MelPop pathology [10]. Selection was conducted on separate and independent flylines and attenuation of pathology in all flylines observed; however it was shown that selection had acted upon the host genome not *w*MelPop [10]. To conclusively determine the outcome of selection in Chrostek and Teixeira 2015 paper would require transfer of selected *w*MelPop into an unselected host background either via backcrossing or microinjection.

In their commentary, Chrostek and Teixeira suggest that non-controlled *w*MelPop *Drosophila* stocks should be a heterogeneous population that is made up of low and high-copy number variants–we agree with that assumption. Based on that assumption, a simulation model of Octomom copy number variation over developmental time is presented, and Octomom copy number is predicted to fluctuate in a similar fashion to that described in our own study. In their commentary Chrostek and Teixeira conclude from this model that “flies carrying *w*MelPop with high Octomom copy numbers die faster and, therefore, at later time points these *Wolbachia* are depleted from the pool of total *w*MelPop and mean Octomom copy number decreases.” The model does not account for a reduction of absolute lifespan of *w*MelPop infected insects when compared to *w*MelCS or uninfected controls. If Octomom amplification is linked to intensity of pathology then variants with a single copy of the Octomom locus should live as long as non-pathogenic strains of *Wolbachia*.

Both studies have highlighted the dynamic nature of the Octomom locus, across generational and developmental time. Our study has shown Octomom amplification is not linked to either the intensity of pathology nor over-replication of *w*MelPop. We conclude that at this point in time there is no clear genetic determinate of *w*MelPop pathology and that further studies of *w*MelPop and related strains are required.

## Supporting information

S1 Fig Octomom copy numbers in *w*MelCS-infected flies. Mean Octomom copy number relative to a single copy *w*MelCS gene in 1, 5 and 15-day old adult *Drosophila* reared at 21°C (black-shaded circles) or 24°C (grey-shaded circles) as determined by qPCR. Flies were reared at 24°C from embryo to eclosion. Days refer to adult fly age post eclosion. Grey bars represent median of n = 6 samples. No significance difference was observed (*F*(2, 27) = 3.35, *p* = 0.87).
(TIFF)

S1 Data Raw density data. Raw *Wolbachia* density data for flies infected with *w*MelCS reared at different temperatures and across different time points.
(XLSX)

## References

[1] Bordenstein SR, Bordenstein SR. Eukaryotic association module in phage WO genomes from Wolbachia. Nat Commun. 2016;7:13155 doi: 10.1038/ncomms13155 ; PubMed Central PMCID: PMCPMC5062602. 27727237 10.1038/ncomms13155 PMC5062602

[2] Bordenstein SR, Reznikoff WS. Mobile DNA in obligate intracellular bacteria. Nature Reviews Microbiology Nat Rev Micro. 2005;3(9):688–99. 10.1038/nrmicro1233 16138097

[3] Brownlie JC, O'Neill SL. Wolbachia genomes: insights into an intracellular lifestyle. Current biology: CB. 2005;15(13):R507–9. doi: 10.1016/j.cub.2005.06.029 . 16005284 10.1016/j.cub.2005.06.029

[4] Chrostek E, Marialva MS, Esteves SS, Weinert LA, Martinez J, Jiggins FM, et al Wolbachia variants induce differential protection to viruses in Drosophila melanogaster: a phenotypic and phylogenomic analysis. PLoS genetics. 2013;9(12):e1003896 doi: 10.1371/journal.pgen.1003896 ; PubMed Central PMCID: PMC3861217. 24348259 10.1371/journal.pgen.1003896 PMC3861217

[5] Chrostek E, Teixeira L. Mutualism Breakdown by Amplification of Wolbachia Genes. PLoS biology. 2015;13(2):e1002065 doi: 10.1371/journal.pbio.1002065 25668031 10.1371/journal.pbio.1002065 PMC4323108

[6] Iturbe-Ormaetxe I, Burke GR, Riegler M, O'Neill SL. Distribution, expression, and motif variability of ankyrin domain genes in Wolbachia pipientis. Journal of bacteriology. 2005;187(15):5136–45. doi: 10.1128/JB.187.15.5136-5145.2005 ; PubMed Central PMCID: PMC1196006. 16030207 10.1128/JB.187.15.5136-5145.2005 PMC1196006

[7] Woolfit M, Iturbe-Ormaetxe I, Brownlie JC, Walker T, Riegler M, Seleznev A, et al Genomic evolution of the pathogenic Wolbachia strain, wMelPop. Genome biology and evolution. 2013;5(11):2189–204. doi: 10.1093/gbe/evt169 ; PubMed Central PMCID: PMC3845649. 24190075 10.1093/gbe/evt169 PMC3845649

[8] Rohrscheib CE, Frentiu FD, Horn E, Ritchie FK, van Swinderen B, Weible MW 2nd, et al Intensity of Mutualism Breakdown Is Determined by Temperature Not Amplification of Wolbachia Genes. PLoS Pathog. 2016;12(9):e1005888 doi: 10.1371/journal.ppat.1005888 ; PubMed Central PMCID: PMCPMC5035075. 27661080 10.1371/journal.ppat.1005888 PMC5035075

[9] McMeniman CJ, Lane AM, Fong AW, Voronin DA, Iturbe-Ormaetxe I, Yamada R, et al Host adaptation of a Wolbachia strain after long-term serial passage in mosquito cell lines. Appl Environ Microbiol. 2008;74(22):6963–9. doi: 10.1128/AEM.01038-08 ; PubMed Central PMCID: PMC2583474. 18836024 10.1128/AEM.01038-08 PMC2583474

[10] Carrington LB, Leslie J, Weeks AR, Hoffmann AA. The popcorn Wolbachia infection of Drosophila melanogaster: can selection alter WOlbachia longevity effects? Evolution. 2009;63(10):2648–57. doi: 10.1111/j.1558-5646.2009.00745.x 19500146 10.1111/j.1558-5646.2009.00745.x

