# Supplementary figures and images for: Response to: Comment on Rohrscheib et al. 2016 "Intensity of mutualism breakdown is determined by temperature not amplification of Wolbachia genes"
Source: PLoS Pathog. 2017 Sep 11;13(9):e1006521. doi: 10.1371/journal.ppat.1006521 (PMC5593257; doi:10.1371/journal.ppat.1006521)

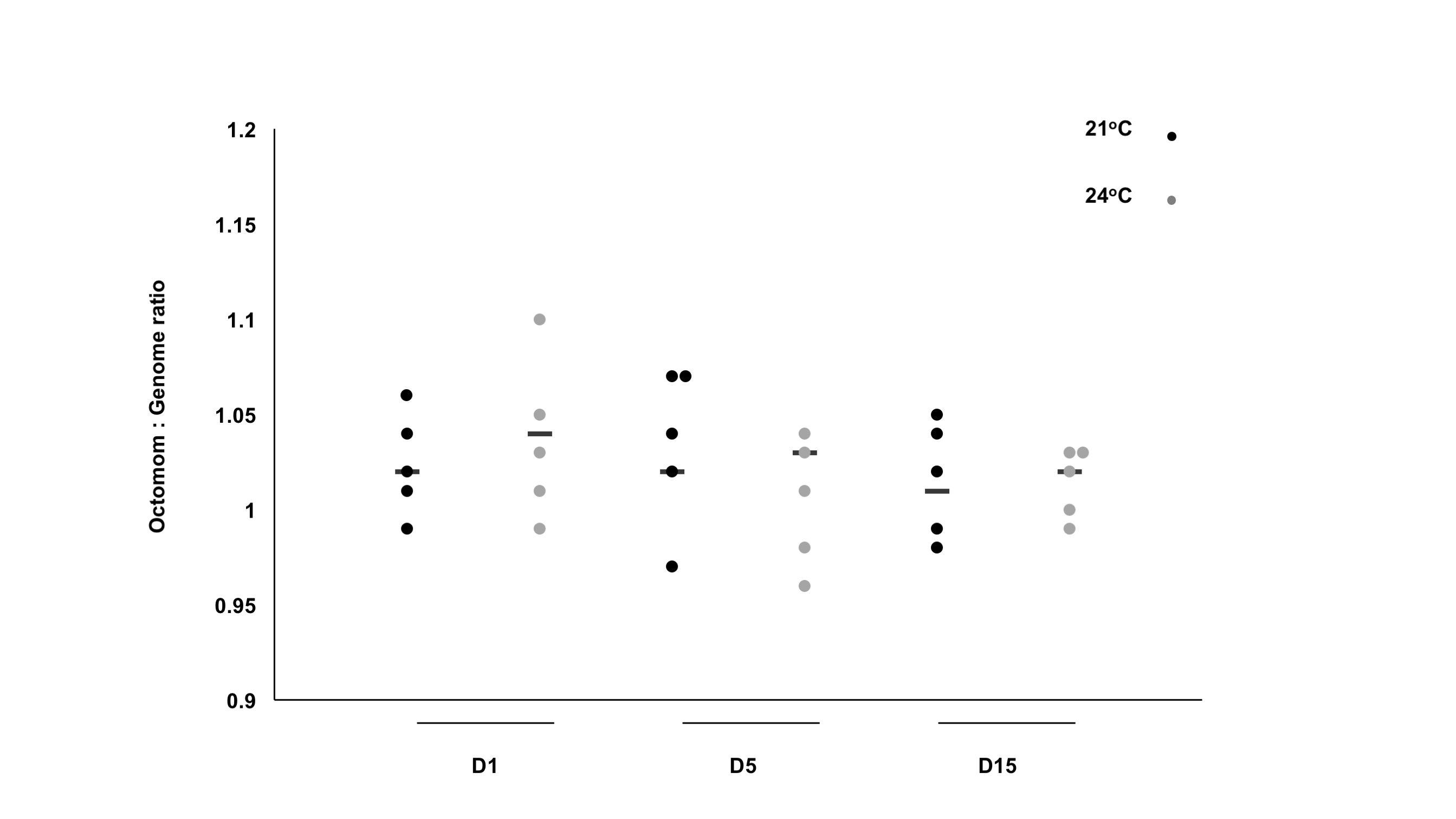

Supplement: S1 Fig — Mean Octomom copy number relative to a single copy wMelCS gene in 1, 5 and 15-day old adult Drosophila reared at 21°C (black-shaded circles) or 24°C (grey-shaded circles) as determined by qPCR. Flies were reared at 24°C from embryo to eclosion. Days refer to adult fly age post eclosion. Grey bars represent median of n = 6 samples. No significance difference was observed (F(2, 27) = 3.35, p = 0.87). (TIFF) [file ppat.1006521.s001.tiff]
